# Endotracheal suctioning in intubated newborns: an integrative literature review

**DOI:** 10.5935/0103-507X.20150048

**Published:** 2015

**Authors:** Roberta Lins Gonçalves, Lucila Midori Tsuzuki, Marcos Giovanni Santos Carvalho

**Affiliations:** 1 Undergraduate Program in Physical Therapy, Faculdade de Educação Física e Fisioterapia, Universidade Federal do Amazonas - Manaus (AM), Brazil.; 2 Residency Program in Physical Therapy in Neonatal Intensive Care, Universidade Federal do Amazonas - Manaus (AM), Brazil.; 3 Physiotherapy Service, Maternidade Balbina Mestrinho, Secretaria de Estado de Saúde do Amazonas - Manaus (AM), Brazil.

**Keywords:** Suction/methods, Respiration, artificial, Infant, newborn

## Abstract

Evidence-based practices search for the best available scientific evidence to support problem solving and decision making. Because of the complexity and amount of information related to health care, the results of methodologically sound scientific papers must be integrated by performing literature reviews. Although endotracheal suctioning is the most frequently performed invasive procedure in intubated newborns in neonatal intensive care units, few Brazilian studies of good methodological quality have examined this practice, and a national consensus or standardization of this technique is lacking. Therefore, the purpose of this study was to review secondary studies on the subject to establish recommendations for endotracheal suctioning in intubated newborns and promote the adoption of best-practice concepts when conducting this procedure. An integrative literature review was performed, and the recommendations of this study are to only perform endotracheal suctioning in newborns when there are signs of tracheal secretions and to avoid routinely performing the procedure. In addition, endotracheal suctioning should be conducted by at least two people, the suctioning time should be less than 15 seconds, the negative suction pressure should be below 100 mmHg, and hyperoxygenation should not be used on a routine basis. If indicated, oxygenation is recommended with an inspired oxygen fraction value that is 10 to 20% greater than the value of the previous fraction, and it should be performed 30 to 60 seconds before, during and 1 minute after the procedure. Saline instillation should not be performed routinely, and the standards for invasive procedures must be respected.

## Abstract (pt)

A prática baseada em evidências se baseia na busca da melhor evidência científica disponível para fundamentar a solução de um problema e a tomada de decisão. Devido à complexidade e à quantidade de informações na área da saúde, há necessidade de integração dos artigos científicos de boa qualidade metodológica disponíveis partindo da revisão da literatura. Mesmo a aspiração endotraqueal sendo o procedimento invasivo mais realizado em recém-nascidos intubados em unidades de terapia intensiva neonatais, poucos são os estudos brasileiros de boa qualidade metodológica que fundamentam essa prática, não havendo consenso ou padronização da técnica nacionalmente. Em virtude do exposto, o objetivo do estudo foi revisar os estudos secundários sobre o assunto para estabelecer recomendações sobre a aspiração endotraqueal em recém-nascidos intubados, favorecendo a adoção do conceito de boas práticas na realização desse procedimento. Para tal, foi realizada uma revisão integrativa da literatura. A recomendação deste estudo é de que a aspiração endotraqueal em recém-nascidos seja realizada apenas quando houver sinais de secreção traqueal, não devendo ser realizada rotineiramente; que seja realizada por, no mínimo, duas pessoas; que o tempo de aspiração seja inferior a 15 segundos e a pressão de sucção inferior a 100mmHg negativos; que a hiperoxigenação não seja utilizada de maneira rotineira. Se indicada, é recomendada a oxigenação com fração inspirada de oxigênio 10 - 20% maior que a anterior, 30 - 60 segundos antes, durante e 1 minuto após o procedimento. Não deve ser realizada a instilação de solução salina rotineiramente e as normas para procedimentos invasivos devem ser respeitadas.

## INTRODUCTION

Evidence-based practices encourage the use of research results in clinical practice, and they are based on the search for the best available scientific evidence to support problem solving and decision making. Because of the quantity and complexity of information related to health care, the available evidence must be integrated through the performance of literature reviews. An integrative literature review represents a research method that is used in evidence-based practice because it allows for the incorporation of evidence into clinical practice. This method aims to gather and synthesize research findings on a defined topic or issue in a systematic and orderly manner to contribute to the deepening of knowledge on the studied subject. Although endotracheal suctioning in intubated newborns undergoing mechanical ventilation (MV) is the most invasive procedure performed in neonatal intensive care units (NICU)^(1-13)^ in Brazil, this procedure is not grounded in the best scientific evidence available.

Newborns in the NICU routinely require MV, which may be non-invasive through an interface connecting the individual to the ventilator or invasive (the most common choice in NICUs) through an inserted endotracheal tube (ETT) connecting the individual to the ventilator.^(1-6)^ The presence of the ETT leads to increased mucus production as a result of mild irritation generated in the airway mucosa, and it also impairs the ability to mobilize and expectorate secretions by suppressing the appropriate mucociliary mechanism and impairing the cough reflex, thus requiring frequent endotracheal suctioning to prevent secretion accumulation and airway obstruction.^(4)^ In newborns, ETT have small internal diameters, which increases the difficulty of the procedure and increases the risk of complications.^(2-4)^ ETT suctioning with internal diameters ≤ 4mm can cause an immediate reduction in dynamic pulmonary compliance and reduce the expired tidal volume (TV), regardless of lung pathology.^(4)^ Moreover, inefficient suctioning can result in the obstruction of the ETT, the need for re-intubation and atelectasis, and a reduction in ventilation and oxygenation.^(2)^

Endotracheal suctioning is a manual mechanical technique for the removal of secretions from individuals who cannot properly remove pulmonary, tracheobronchial and/or oropharyngeal secretions. This procedure is routinely used in patients who require artificial airways and MV,^(1-6)^ and it consists of introducing a sterile flexible tube through the airway and applying sub-atmospheric pressure at the time of its removal to suck out the secretions.^(1-19)^ Despite the clinical importance of this technique, few methodologically sound Brazilian studies have been performed on this subject, and there is a lack of national consensus and clinical standardization regarding various points of the technique. Certain Brazilian services perform endotracheal suctioning in newborns according to criteria based on institutional routines or even the professional’s individual practice, which increases the chance of complications.

Therefore, the purpose of this study was to review secondary studies on the subject of endotracheal suctioning to establish recommendations for intubated newborns and encourage the adoption of best-practice concepts when conducting this procedure and the incorporation of scientific evidence into clinical practice.

## METHODS

This paper presents an integrative review of secondary studies (guidelines and systematic reviews, with or without meta-analysis) published in English between 2000 and 2013. The goal was evaluate the open system endotracheal suctioning technique in human newborns undergoing invasive MV. The Cochrane, PEDro and PubMed databases were searched using the keywords “infant” and “newborn” and the following related terms identified in the Medical Subject Headings (MESH) database: “infants,” “newborn infant,” “newborn infants,” “newborns,” and “neonate.” In addition, these terms were combined with the intervention “suction” and the specific correlates identified in MESH: “suctions,” “aspiration,” “mechanical,” “aspirations,” “mechanical aspiration,” “mechanical aspirations,” “drainage,” “drainages,” “suction drainage,” and “suction drainages.” The searches of the Cochrane and PubMed databases were performed combining terms using the Boolean operators “AND” and “OR.” The PEDro database did not allow the use of both Boolean operators at the same time; therefore, this database was searched by an individual combination of terms and their correlates.

The selection of articles was based on the combination of patient-infant or -newborn and intervention-suction for newborns undergoing invasive MV, and the following clinical issues were addressed: frequency, duration, probe diameter, hyperoxygenation, negative suction pressure, saline instillation, number of repetitions, extraction time, absolute contraindications and biosecurity standards. Articles that explicitly defined the evidence level of each analyzed parameter were retained according to the evidence level and recommendation grade proposed by the Cardiology Commission (*Comissão de Cardiologia*); these grades are based on evidence from the Brazilian Society of Cardiology (*Sociedade Brasileira de Cardiologia*) and Brazilian Medical Association (*Associação Médica Brasileira*).

## RESULTS

The search resulted in 93 publications (57 from Cochrane, 19 from PEDro and 17 from PubMed), which were analyzed according to the desired criteria. Of these, 89 articles were excluded for not addressing endotracheal suctioning in newborns or because they were not systematic reviews with or without meta-analyses or guidelines. Of the included studies, one was available on both the PEDro and PubMed databases (duplicate). Thus, four articles (four guidelines) remained, and they were included and analyzed in this integrative review as shown in table 1. The proposed results and the evidence level of each study are shown in table 2. Studies that were not consistent with the established search criteria, such as studies in Portuguese, but that were relevant to the subject under discussion were analyzed and used as a basis for discussion; however, they did not contribute to this study’s recommendations.

**Table 1:** Relevant aspects of the evaluated articles

**Title, year of publication and source**	*Nasotracheal Suctioning - 2004 Revision & Update,* 2004, *Respiratory* Care^(10)^	ERNBG Guideline - Suction February 2006. Review: February 2006 Eastern Regional Neonatal Benchmarking Group Suctioning Guideline^(9)^	Evidence-based guideline for suctioning the intubated neonate and infant, 2009, *Neonatal Network*^(2)^	*Endotracheal suctioning of mechanically ventilated patients with artificial airways,* 2010,* Respiratory Care*^( 6)^
**Hyperoxygenation before, during and after the procedure**	Not stated	Pre-oxygenation should not be performed unless SpO_2_ has dropped	Data regarding hyperoxygenation in NB are limited. Therefore, care must be taken when using oxygenation on this population	Pre-oxygenation is suggested if the patient presents a clinically relevant reduction in SpO_2_ with suctioning
				**Evidence level 2B**
				In NB a 10% increase in FiO_2_ is recommended before suctioning, especially in hypoxemic NB
				Hyperoxygenation should be maintained for at least 1 minute after suctioning, especially in hypoxemic patients
**Characteristics of the suctioning probe**	The probe should be sterile, flexible, with various lateral orifices and one frontal one	The probe must be measured prior to the procedure to ensure that the probe does not overshoot the end of the ETT	The diameter of the probe should be less than 50% of the ETT diameter	The probe diameter should not occlude more than 70% of the light in the ETT in small children
			**Evidence level V**	**Evidence level 2C**
		The diameter of the probe should not exceed 50% of the internal diameter of the ETT	Probes bigger than 6 F should not be used for suctioning in a 2.5 ETT	
			**Evidence level V**	
**Suctioning time**	Must be limited to 15 sees.	Must be limited to 10 - 15 secs.	Must be limited to 15 secs.	Must be limited to 15 secs.
			**Evidence level V**	**Evidence level 2C**
**Negative suction pressure**	60 - 80 mmHg	50 - 100mmHg	Must not exceed 100 mmHg	80 - 100mmHg.
			**Evidence level V**	
			Suctioning should be applied only when removing the probe	
			**Evidence level 3**	
**Saline instillation**	Not stated	Use limited to NB whose secretions may obstruct airways	Should not be performed routinely	Should not be performed routinely
			**Evidence level IV**	**Evidence level 2C**
**Number of repetitions**	There is controversy regarding the excessive use of this procedure	Normally, one or two attempts are sufficient for cleaning secretions	Should not exceed three repetitions when suctioning	Not stated
**Suctioning time**	When clinically indicated	When the need for the procedure is identified	When the need for the procedure is identified	Only when there is secretion and not routinely
			**Evidence level I**	**Evidence level 1C**
**Absolute contraindication**	Not stated for intubated NB	Not stated	Not stated	There is no absolute contraindication
** Biosafety standards**	CDC guidelines for standard precautions should be respected	Not stated	Not stated	CDC guidelines for standard precautions should be respected

SpO_2_ - peripheral capillary oxygen saturation; FiO_2_ - inspired oxygenation fraction; NB - Newborn; ETT - endotracheal tube; CDC - Center for Disease Control and Prevention.

**Table 2:** Recommendations for endotracheal suctioning in intubated newborns

**Clinical issue**	**Recommendation**	**Articles in agreement (%)**	**Evidence level**
Hyperoxygenation	Hyperoxygenation should not be incorporated into the suctioning routine. If there is a drop in SpO_2_ with suctioning, hyperoxygenation should be established by increasing the FIO_2 _value 10 to 20% above that prior to suctioning 30 to 60 seconds before, during and 1 minute after the procedure	50	2B
Suctioning probe diameter	Probe diameter must not exceed 50% of ETT diameter	50	2C
Suctioning duration	Must not exceed 15 seconds	100	2C
Negative suction pressure	Must be between 50 and 100mmHg	75	3C
Saline instillation	Must not be performed routinely	75	2C
Number of repetitions	Must not exceed 3 repetitions. The NB should be connected to the ventilator between suctions	25	-
Suctioning time	Suctioning must not be performed routinely and should only be performed where clinically indicated. Clinical indications primarily include lung auscultation (coughing, coarse or reduced breathing) or visible secretions in the ETT, audible secretions, drop in SpO_2 _decreased chest excursion, changes in blood gas values, changes in respiratory rate and/or breathing pattern, bradycardia/tachycardia and/or agitation without other cause, and increased peak pressure on the ventilator	100	1C
Biosafety standards	CDC guidelines should be respected, including the following: protection of the professional's eye, nose and mouth protection with the use of face mask and goggles, use of apron and sterile gloves, and performance of hand hygiene before and after performing the procedure. To increase safety, the procedure must be performed by at least two people	50	-
Monitoring	The following variables should be monitored before, during and after the procedure: SpO_2_ skin coloring, respiratory frequency; respiratory pattern; hemodynamic variables (if monitored), such as heart rate, blood pressure, heart rhythm and ICP; suctioned secretion characteristics, such as color, volume, consistency and odor; cough characteristics; ventilatory parameters, such as peak inspiratory pressure and plateau pressure; current volume; flow; exhaled volume; and FiO_2_	100	-

SpO_2_ - peripheral capillary oxygen saturation; FiO_2_ - inspired oxygenation fraction; NB - newborn; ETT - endotracheal tube; CDC - Center for Disease Control and Prevention; ICP - intracranial pressure.

## DISCUSSION

Endotracheal suctioning in intubated newborns undergoing MV is a procedure that is routinely performed by physiotherapists, doctors, nurses, and also by nursing technicians in Brazil as a component of the resuscitation procedure and bronchial hygiene therapy.^(1-20)^ The goal is to maintain airway patency and facilitate ventilation and oxygenation.^(1-6)^ However, this technique has specific indications and adverse effects. Proper standardization, clear indications for use and definitions of the procedure all serve to minimize complications.

Endotracheal suctioning is an important element of care for newborns admitted to the NICU because most of these patients require invasive MV and repeated and frequent suctioning for the removal of tracheal secretions.^(2,4,8,9,12,20-29)^ According to the American Association of Respiratory Care (AARC), proper suctioning in intubated individuals improves gas exchange and respiratory sounds; decreases airway resistance and peak inspiratory pressure of the ventilator; improves dynamic compliance; increases the TV release when in limited pressure ventilation mode; and improves arterial blood gas and oxygen saturation (SpO_2_) values.^(6,10)^ However, many institutions attempt to maintain airway patency in intubated individuals by adopting protocols that include the routine use of endotracheal suctioning without evaluating whether the procedure is necessary. These protocols are primarily based on the care ritual than on evidence of the clinical need for suctioning.

All of the studies included in this review described the technique as mandatory, i.e., that it should be performed whenever necessary because the accumulation of tracheobronchial secretions may impair ventilation and oxygenation; lead to ETT occlusion, atelectasis and increased respiratory work; and predispose the individual to pulmonary infection.^(2,3,6,8-10,15-28)^ However, one of the most controversial issues regarding endotracheal suctioning in neonates is the precise time and frequency at which the technique should be performed on intubated individuals.

The results of this review reveal strong evidence that neonatal endotracheal suctioning should not be routinely performed and should only be undertaken when indicated, which occurs when there are signs of secretion.^(2,6,9,10)^ The endotracheal suction procedure is not considered benign, although peripheral secretions are not removed with this single procedure.^(8)^ Thus, it is important to perform individual evaluations of patients to determine whether suctioning should be performed. The following clinical criteria were considered for suctioning in intubated newborns: visible secretions in the ETT, audible secretions, coarse or decreased breathing sounds, decreased respiratory excursion, change in blood gases, changes in respiratory rate, bradycardia/tachycardia and/or agitation without other cause, increase in peak respiratory pressure and reduction in TV. The need for endotracheal suctioning in newborns is preferably evaluated using auscultation.^(1,5,8,9)^ Therefore, the recommendation of this study is that the decision to perform endotracheal suctioning in newborns should be based on individual evaluations and clinical criteria identifications that indicate the need for suctioning and performing endotracheal suctioning should not be established as part of the routine care of intubated newborns.

There were no absolute contraindications for endotracheal suctioning in newborns, which was most likely because of the mandatory nature of the procedure. Most of the articles did not address this issue and only one study indicated the lack of absolute contraindications for endotracheal suctioning in newborns.^(7)^ In clinical practice, endotracheal suctioning is avoided in newborns 15 to 30 minutes after surfactant administration.^(28)^ In special cases, such as uncontrolled intracranial hypertension, additional measures to control intracranial pressure (ICP) must be adopted prior to endotracheal suctioning in newborns.

The decision regarding the exact time to perform endotracheal suctioning is important because this procedure is not without complications and adverse effects. Several adverse effects of endotracheal suctioning in newborns have been identified, such as hypoxemia, bradycardia, hypotension and reduced SpO_2_.^(19-23)^ These effects have been related to the suctioning of air from the airways and vagal stimulation because of the introduction of the probe and the negative pressure generated in the airway.^(4)^ Complications most commonly reported include trauma, bleeding, mucosal injury, atelectasis because of excessive suctioning of air from the airways, hypertensive peaks as a result of the reflex discharge of the sympathetic nervous system and bronchospasm.^(7)^ The most serious complications are hypoxemia, increased blood pressure, increased ICP and pneumothorax.^(8)^ Evidence has shown that these adverse effects can be minimized or eliminated by proper performance of the technique.^(2)^

Hypoxemia is the complication most often related to endotracheal suctioning in newborns; thus, hyperoxygenation is an important clinical issue. Hyperoxygenation occurs when the inspired oxygen fraction (FiO_2_) is administered at a higher percentage than what was delivered prior to suctioning at values reaching up to 100%.^(2)^ Although hyperoxygenation is widely used in clinical practice to avoid hypoxemia, half of the authors examined in this review suggest that hyperoxygenation should not be conducted routinely but rather when the newborn has a clinically relevant reduction in SpO_2_ during suctioning.^(6,9)^ This recommendation was based on the adverse effects caused by excess oxygen, even when applied for short periods of time, in newborns, such as hypercapnia, atelectasis by absorption, retinopathy of prematurity, alveolar and tracheobronchial changes, lung parenchyma, and especially oxidative stress, which leads to inflammatory responses, particularly in preterm newborns that do not have fully functioning antioxidant mechanisms.^(9,17)^ Although there was a lack of consensus regarding hyperoxygenation in newborns, most of the studies in favor of this procedure agreed that the FiO_2_ rate should remain between 10 - 20% above the level prior to endotracheal suctioning.^(15,16)^ To prevent hypoxemia in newborns, an increase of 20% in FiO_2_ relative to that prior to suctioning is likely as effective as hyperoxygenation with 100% FiO_2_.^(14-16)^ Studies have even suggested that for most newborns, it is only necessary to increase the FiO_2_ value by 10% relative to the pre-suctioning value.^(20-22)^

With regard to whether FiO_2_ should be increased before or after endotracheal suctioning, there was no consensus and not all of the studies reported this variable. In one study, the recommendation was to increase FiO_2_ 30 to 60 seconds before suctioning and 1 minute thereafter.^(6)^ Therefore, the suggestion of this review is that if the newborn’s SpO_2_ falls during endotracheal suctioning, hyperoxygenation at an FiO_2_ value 10 - 20% higher than that prior to suctioning should be performed during the next suctioning and 30 to 60 seconds before the procedure, and this fraction should be maintained during suctioning and 1 minute thereafter. However, SpO_2_ must be monitored in all newborns requiring endotracheal suctioning; therefore, individualized hyperoxygenation parameters should be adopted according to clinical alterations during the procedure.^(8,20-22)^

Another potential complication of hyperoxia in newborns is the flip-flop phenomenon, which refers to a larger than expected drop in partial pressure arterial oxygen (PaO_2_) when FiO_2_ values are reduced to levels prior to endotracheal suctioning. This complication most likely occurs because of the reflex pulmonary vasoconstriction phenomenon. The pulmonary capillaries are sensitive to changes in PaO_2_ and changes in regional relations that increase the right-left shunt, which causes a disproportionate drop in PaO_2_ with reductions in FiO_2_.^(19,24)^ Therefore, according to the Guide to Newborn Health Care (*Atenção à Saúde do Recém-Nascido*) of the Ministry of Health (*Ministério da Saúde*), if the FiO_2_ values are above 60%, the reduction in FiO_2_ should be 10% every 15 to 30 minutes to avoid the flip-flop effect.^(24)^

The procedure duration was correlated with the severity of adverse effects, and longer suctioning time produced a greater risk of damage to the tracheal mucosa and hypoxemia.^(8,13-17)^ The analyzed studies all concluded that the duration of each suction event should not exceed 15 seconds.^(2,6,9,10)^ However, there was a lack of consensus among the analyzed articles regarding the number of repetitions of endotracheal suctioning in newborns. According to Evidence-based guideline for suctioning the neonate and intubated infant, the probe size and negative pressure amount influenced the required number of repetitions.^(2)^ In addition, an increased number of probe insertions during endotracheal suctioning increased the likelihood of complications, such as mucosal trauma, hypoxemia, laryngeal spasm, bronchospasm, necrotizing tracheitis, infection and discomfort, and it also increased the likelihood of barotrauma.^(2,9)^ Therefore, we suggest that the probe insertion frequency should not exceed three repetitions and recommend that an appropriate amount of time should be allowed between suctioning events so that the monitored variables can return to baseline levels. In addition, the newborn should be reconnected to the ventilator at this time.^(2,8,9,10)^ All of the studies concluded that hyperinsufflation, or ventilation with TV higher than that established on the MV, is not recommended in newborns because hyperinsufflation may provoke changes in FiO_2_ that would require the newborn to be reconnected to the respirator.^(8)^ Therefore, the recommendation of this study is to perform the procedure is performed until improvements are observed in lung auscultation or the clinical parameters that led to suctioning. In addition, the procedure should be performed as efficiently as possible so that fewer repetitions are necessary, and a maximum of three repetitions are recommended for endotracheal suctioning in newborns.

According to the AARC and Evidence-based guideline for suctioning the intubated neonate and infant, the procedure is safer when certain variables are monitored before, during and after it is performed. These variables include respiratory sounds; SpO_2_; skin coloring; respiratory rate; breathing pattern; hemodynamic variables (if monitored), such as heart rate, blood pressure, heart rhythm and ICP; aspirated secretion characteristics, such as color, volume, consistency and odor; cough characteristics; ventilatory parameters, such as peak inspiratory pressure and plateau pressure; TV; flow; exhaled volume; and FiO_2_.^(2,10)^ One study mentioned that to reduce changes in cerebral blood flow, the head of the newborn should be positioned to align with the midline.^(9)^ Therefore, the recommendation of this study is to monitor these clinical variables before, during and after the procedure.

Half of the analyzed studies concluded that the diameter of the suctioning probe should not exceed 50% of the diameter of the ETT and recommended using the smallest suction probe capable of properly removing secretions. This recommendation is based on the fact that the suction probe size most likely has an increased influence on lung volume loss relative to negative suction pressure.^(6,8,10)^ The larger the size of the suction probe and more negative the suction pressure, the greater the suctioned gas flow and the more negative the tracheal pressure during ETT suctioning. Thus, for a given diameter of ETT, the suction pressure level transmitted to the airway is determined by a combination of suction tube size and suction pressure.^(6,8,10)^

Negative suction pressure can damage the tracheobronchial mucosa by invagination caused by the probe orifice, which may rupture the capillaries.^(13)^ The evaluated studies recommended that suction pressure should be checked by occluding the end of the suction probe before starting the procedure and ensuring that the suction pressure is as low as possible while still being able to remove secretions because negative tracheal pressure during suction is directly proportional to the applied pressure.^(4)^ Negative pressures of 200mmHg with continuous and intermittent suctioning are capable of damaging the mucosa.^(4,9,13)^ All of the articles analyzed in this study suggested that negative suction pressure in newborns should not exceed 100mmHg.^(2,6,9,10)^ One study suggested subatmospheric pressures under 80mmHg. Therefore, the recommendation of this review is to apply suction pressure between - 50 to -100mmHg (8 - 10kPa) and avoid exceeding 100mmHg negative.

Saline instillation during endotracheal suctioning is a controversial topic in pediatrics, especially in neonatology. This practice is widely used in Brazilian ICU and corresponds to the instillation of saline aliquots (typically between 1 and 5ml) in the ETT before or during insertion of the suctioning probe. This procedure is based on the premise that saline facilitates the mobilization, release and removal of secretions and suctioning tubes lubricated with saline will reduce friction with the ETT and prevent triggering of the cough reflex.^(6)^ However, saline instillation increases the likelihood of cardiac arrhythmia, hypoxemia, atelectasis, bronchospasm, infection, mucosal and respiratory tract cilia trauma and increases ICP.^(2)^ In Ridling et al.’s study, saline instillation had an adverse effect on the reduction of SpO_2_ in the first and second minute after suctioning; however, the adverse effects were absent 10 minutes after the procedure.^(27)^ According to Walsh et al., insertion of the suctioning tube can dislodge thousands of bacteria, and a saline jet may increase the risk of distributing these bacteria to the lungs, thus increasing the incidence of MV-associated pneumonia.^(15)^ Consensus was observed in most of the analyzed articles that saline instillation should not be performed routinely.^(6,8,10)^ Therefore, the recommendation of this study is to avoid the routine performance of saline instillation except in special situations, such as in the case of thick secretions and “secretion plugs” that would be impossible to remove without such a procedure.

Consensus was observed in half of the analyzed studies that the Center for Disease Control and Prevention (CDC) guidelines for standard precautions in invasive procedures must be respected to reduce instances of pneumonia associated with the procedure.^(6,10)^ The remaining analyzed studies did not address this issue. In addition, certain studies argued that endotracheal suction is a procedure that should be performed by two people, thereby increasing safety of the procedure.^(6,8,10)^ On the National Health Surveillance Agency’s (*Agência Nacional de Vigilância Sanitária* - ANVISA) website, references were not found regarding the biosafety standards for endotracheal suctioning or for invasive procedures. Therefore, with regard to the bio-security standards for the performance of endotracheal suctioning in intubated newborns, the recommendation of this review is to follow the CDC guidelines, which advocate protecting the professional’s eyes, nose and mouth using a face mask and goggles, wearing an apron and sterile gloves, and performing hand hygiene before and after the procedure.^(29)^ An eye protector may also be placed on the newborn to prevent mucosa contamination. A further recommendation is for the procedure to be performed by at least two people.^(8,9)^

The implementation of guidelines based on scientific evidence can reduce the risks associated with endotracheal suctioning in newborns. These risks include physiological changes, pneumonia, tracheal damage, hyperoxygenation- and hypoxia-related changes, stress and discomfort. Although international guidelines are available, Brazilian recommendations are lacking, and the procedures adopted in Brazilian NICUs may differ from foreign procedures because of various differences in equipment, sociodemographic parameters, dispensed care, etc. Thus, recommendations and guidelines for this procedure in Brazil can contribute to improving the outcome of newborns in Brazilian NICUs.

Methodologically sound Brazilian endotracheal suctioning studies are scarce, especially in neonatology; therefore, generalizing the available data is difficult. The main limitations of this study were the small number of included articles, poor methodological descriptions and the absence of national secondary studies that address this issue. Additional studies are needed to increase the safety of the procedure and ground the technique in more consistent scientific evidence.

## RECOMMENDATIONS

Based on the analyzed studies, the recommendation of this integrative review is to only perform endotracheal suctioning in intubated newborns when there are clinical signs of tracheal secretions, which are primarily evaluated by the presence of snoring or decreased breathing sounds on auscultation. This procedure should not be routinely performed to prevent airway obstruction. The suction time should not exceed 15 seconds, and the negative pressure must not exceed 100mmHg. Hyperoxygenation should not be used routinely and is only indicated when the baby has a clinically significant reduction in peripheral oxygen saturation during suctioning. When required to reduce hypoxemia, pre-oxygenation is recommended 30 to 60 seconds before, during and 1 minute after endotracheal suctioning by applying an inspired oxygen fraction that is 10 to 20% higher than what was used in the previous procedure. Saline instillation should not be routinely performed. Moreover, the Center for Disease Control and Prevention standards for invasive procedures must be respected during the procedure, the procedure must be conducted by at least two professionals, a maximum of three probe insertions should be performed, with a return to the ventilator between suctionings.
